# Camptothein-Based Anti-Cancer Therapies and Strategies to Improve Their Therapeutic Index

**DOI:** 10.3390/cancers17061032

**Published:** 2025-03-20

**Authors:** Jue Gong, Wenqiu Zhang, Joseph P. Balthasar

**Affiliations:** Department of Pharmaceutical Sciences, University at Buffalo, Buffalo, NY 14214, USAwenqiuzh@buffalo.edu (W.Z.)

**Keywords:** camptothecin, cancer, antibody–drug conjugate, targeted therapy

## Abstract

Camptothecin and its derivatives (CPTs) are potent antineoplastic agents that exert their effects by inhibiting DNA topoisomerase I, leading to apoptosis during cell proliferation. Since their discovery in the 1960s, CPTs have faced challenges such as low water solubility, pH-dependent lactone ring instability, and severe off-target toxicities. Despite extensive research, only two CPTs, irinotecan and topotecan, have received health authority approval. Ongoing clinical trials continue to explore the use of CPTs in combination with targeted therapies and immunotherapies to expand their clinical use. Drug delivery systems, including liposomes and antibody–drug conjugates (ADCs), have significantly enhanced the therapeutic index of CPTs. Liposomal irinotecan (Onivyde^®^, Ipsen, Paris, France) and two ADCs delivering CPT payloads, trastuzumab deruxtecan (Enhertu^®^, Daiichi Sankyo, Tokyo, Japan) and sacituzumab govitecan (Trodelvy^®^, Gilead Sciences, Inc., Foster City, CA, USA), have demonstrated substantial efficacy and safety. There is promise that novel strategies such as inverse targeting and co-dosing with anti-idiotypic distribution enhancers may expand the utility of CPT ADCs. This review highlights CPT therapies in clinical use and discusses approaches to further enhance their therapeutic selectivity.

## Simple Summary

Camptothecin has been widely investigated over the past decades as an anti-cancer agent. However, its applications have been limited by poor water solubility, low stability, and substantial toxicity. Even for camptothecin derivatives with improved physicochemical properties, toxicity remains a major challenge to their development. In this review, we discuss strategies that have been employed, or that are under development, to improve the therapeutic index of camptothecin-based chemotherapeutics.

## 1. Introduction

Camptothecin (CPT) is a potent antineoplastic compound originally extracted from the Chinese tree Camptotheca acuminata. It was first introduced by Wall et al. in the 1960s, and since then, CPT and its derivatives (collectively, CPTs) have been extensively studied as chemotherapeutics for the treatment of various types of cancer [1,2,3]. The mechanism of action of CPTs was discovered in the late 1980s [4]; CPTs exert their antineoplastic effect by inhibiting the enzyme DNA topoisomerase I, interfering with DNA replication, and resulting in apoptosis during cell proliferation [5]. This mechanism grants CPTs a broad anti-cancer spectrum but also leads to their non-selective destruction of healthy proliferating cells. The most common clinically reported adverse effects associated with CPTs are gastrointestinal toxicities, neutropenia, and myelosuppression [6].

**Figure 1:**
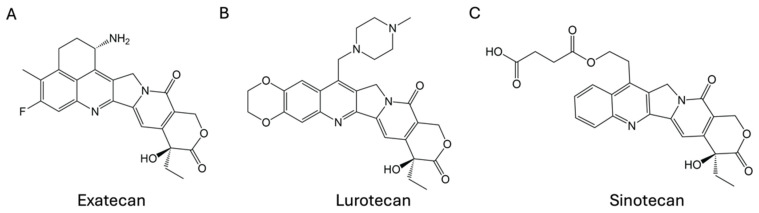
Structures of (**A**) exatecan, (**B**) lurtotecan, and (**C**) sinotecan.

In addition to severe treatment-emergent adverse events in patients, the development and application of CPTs have also been limited by the low water solubility and instability of the active lactone ring structure under physiological conditions. Therefore, chemical modifications of CPT have led to the development of hundreds of derivatives aimed at improving solubility, stability, and safety [7,8,9]. Despite extensive studies over the past few decades, only two CPT analogs, topotecan and irinotecan, have been approved by the US Food and Drug Administration (FDA) [10,11,12]. Topotecan is primarily used for the treatment of ovarian and small-cell lung cancers, while irinotecan is widely used for colorectal cancer. Water-soluble derivatives such as exatecan, lurtotecan, and sinotecan (Figure 1) have been tested in clinical trials but were discontinued mainly due to unmanageable toxicity and insufficient efficacy [13,14,15].

These limitations necessitate the development of novel approaches to enhance the safety and efficacy of CPTs. Drug delivery systems such as liposomes, polymeric nanoparticles, and antibody–drug conjugates (ADCs) have been successfully employed for the targeted delivery of CPTs [16,17]. For example, liposomal irinotecan (Onivyde^®^, Ipsen, Paris, France) was approved in combination with 5-fluorouracil and leucovorin in 2015 for the treatment of metastatic pancreatic cancer and recently moved to the front line in 2024. Two ADCs that deliver CPTs, trastuzumab deruxtecan (T-DXd, Enhertu^®^, Daiichi Sankyo, Tokyo, Japan) and sacituzumab govitecan (Trodelvy^®^, Gilead Sciences, Inc., Foster City, CA, USA), gained global approval in succession as anti-cancer treatments [18,19]. Other approaches, such as inverse targeting strategies and co-dosing ADCs with anti-idiotypic distribution enhancers, have also been investigated to work in parallel with the drug delivery system to further enhance the safety and efficacy CPT-based therapies.

**Figure 2:**
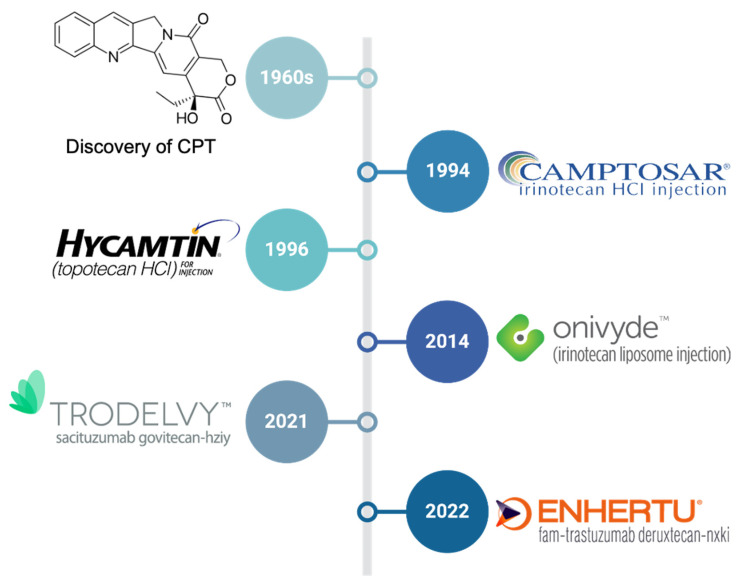
The timeline of the discovery of camptothecin (CPT) and approval of CPT-based chemotherapeutics.

Here, we provide a brief overview of the approved CPT-based chemotherapeutics (Figure 2) and discuss novel strategies for improving the therapeutic index of CPTs.

## 2. Small-Molecule CPT Derivatives

### 2.1. Irinotecan (CPT-11)

**Figure 3:**
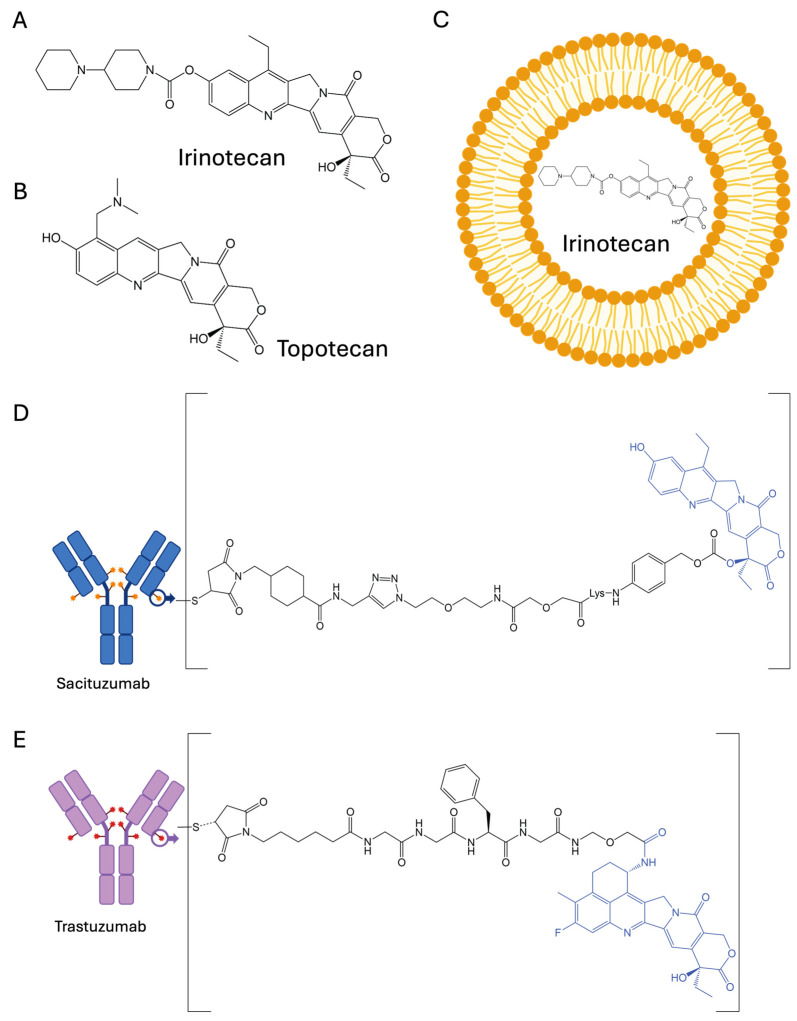
The structures of approved CPT-based chemotherapeutics: (**A**) irinotecan, (**B**) topotecan, (**C**) liposomal irinotecan, (**D**) sacituzumab govitecan, and (**E**) trastuzumab deruxtecan.

Irinotecan (Campstar^®^, Pfizer, Inc., New York City, NY, USA), also known as CPT-11 (Figure 3A), was the first clinically evaluated water-soluble CPT derivative [20,21]. It was initially approved in Japan in 1994, followed by the United States in 1996, primarily for the treatment of metastatic colorectal cancer.

**Figure 4:**
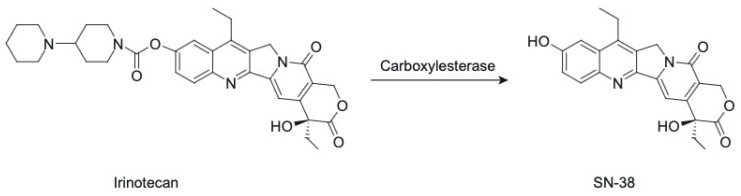
The conversion of irinotecan to SN-38.

Irinotecan functions as a prodrug, as its metabolic product SN-38 is more than 1000-fold more potent in cytotoxicity compared to the parental CPT-11 [22]. The metabolism of CPT-11 involves its conversion to SN-38 primarily by the enzyme carboxylesterase (Figure 4), found in the liver and intestines, and the subsequent glucuronidation and detoxification of SN-38 by the UGT1A1 enzyme [23]. Genetic polymorphisms in UGT1A1 were found to play an important role in SN-38 metabolism and, therefore, CPT-11 toxicity [24]. This complex metabolic pathway contributes to the observed high inter-subject variability in the pharmacokinetics of CPT-11 and SN-38, which ultimately translates into complications in safety and efficacy exposure–response relationships in patients.

The most common dose-limiting toxicities (DLTs) of CPT-11 are gastrointestinal toxicity, including severe acute and delayed-onset diarrhea, and myelosuppression, particularly neutropenia [11,12,25]. Clinically, acute and delayed diarrhea is managed through atropine and high-dose loperamide regimens, while myelosuppression requires dose adjustments and supportive care with colony-stimulating factors. In addition, genetic testing for UGT1A1 polymorphisms has been applied to guide dosing to minimize toxicity risks [26,27].

CPT-11 remains one of the most important chemotherapeutic agents in oncology treatment. It has been moved to the first-line therapy in combination with 5-fluorouracil and leucovorin for patients with metastatic colon cancer [28]. Ongoing clinical trials are exploring its use in combination with newer targeted therapies and immunotherapies, such as bevacizumab (Avastin^®^, Roche, South San Francisco, CA, USA) for recurrent glioblastoma, nivolumab (Opdivo^®^, Bristol-Myers Squibb Company, Princeton, NJ, USA) for gastric cancer, and pembrolizumab (Keytruda^®^, Merck, Rahway, NJ, USA) for grade neuroendocrine cancer [29,30,31]. Additionally, oral formulations like Oncoral^®^ (Ascelia, Malmö, Sweden) are being developed to enhance patient convenience and compliance and have demonstrated activity in early clinical trials for solid tumors [32].

### 2.2. Topotecan

Following FDA approval of topotecan (Hycamtin^®^, Novartis, Basel, Switzerland) (Figure 3B) in 1996, its application as a chemotherapeutic has been extended to the front-line treatment of small-cell lung cancer (SCLC) and second-line treatment of recurrent ovarian cancer and cervical cancer [10,33]. The chemical modification of topotecan at the lactone ring enhances its solubility at physiological pH compared to parental CPT, making it more suitable for intravenous administration. Later, in 2007, an oral formulation of topotecan capsules was approved as the only oral single-agent chemotherapy for the treatment of SCLC [34].

In contrast to irinotecan, which functions as the prodrug for SN-38, topotecan is administered in its active form, directly inhibiting TOP1 to exert its anti-cancer effect. This also results in more predictable pharmacokinetics, safety, and efficacy of topotecan in patients [35]. The major adverse effects of topotecan include neutropenia, thrombocytopenia, and anemia. Several reports have indicated that the hematological toxicities of topotecan are dose-level- and dose-frequency-dependent [36,37]. The standard regimen for topotecan involves intravenous infusion over five consecutive days, repeated every 21 days. Reducing the initial dose and an alternative weekly dosing schedule have lessened topotecan toxicities while maintaining efficacy [38,39].

Topotecan’s manageable toxicity profile provides opportunities for combination therapy. Ongoing clinical trials are exploring its combination with other cytotoxic and targeted therapies, such as intravenous topotecan and oral erlotinib for refractory solid tumors and the combination of tazemetostat with topotecan and pembrolizumab for SCLC [40,41].

## 3. Application of Delivery Systems to Enhance Safety and Efficacy

### 3.1. Liposomes and Polymeric Nanoparticles

Liposomes were first described in the mid-1960s and have been extensively studied as delivery systems for anti-cancer chemotherapeutics, including doxorubicin, paclitaxel, and CPT derivatives [42,43]. Liposomes are self-assembled nanoscale vesicles composed of phospholipid bilayers with an aqueous core. The unique structure allows the loading of small molecules that are either hydrophobic into the lipid bilayer or hydrophilic into the aqueous core.

Liposomal drug delivery may protect encapsulated drug molecules from structural change due to physiological conditions, control the release of drug molecules over time, and enhance the drug’s systemic half-life [44]. Importantly, liposomes leverage the enhanced permeability and retention (EPR) effect, which allows them to preferentially accumulate in tumor tissues due to the immature and leaky nature of tumor vasculature, as well as impaired lymphatic drainage at the tumor site [45]. EPR provides a means of passive targeting of liposomal preparations of chemotherapeutics to tumors, improving the therapeutic index of chemotherapy.

Nal-IRI (Figure 3C), a liposomal formulation of irinotecan (Onivyde^®^), was first approved by the FDA in 2014 for the treatment of metastatic pancreatic ductal adenocarcinoma (mPDAC) following gemcitabine-based therapy [46]. It is now considered to be the front-line treatment of mPDAC in combination with oxaliplatin, fluorouracil, and leucovorin [47]. Nal-IRI releases irinotecan in the tumor microenvironment, where irinotecan is converted into its active metabolite SN-38 [48]. This targeted and controlled process results in a less fluctuating PK profile of SN-38 in the systemic circulation, thus improving the safety profile compared to traditional irinotecan [49]. As observed in patients, nal-IRI reduces the incidence and severity of gastrointestinal toxicity, particularly delayed-onset diarrhea, a major DLT of irinotecan [50]. In addition, based on population PK analysis, genetic polymorphism of the UGT1A1 enzyme was not found to be a significant predictor of SN-38 concentration following nal-IRI administration, aligned with the observation of reduced inter-subject variability of nal-IRI, relative to CPT-11, in clinical trials [51].

Attempts have been made to encapsulate other CPT derivatives such as SN-38 and topotecan. Liposomal LE-SN-38 failed in a phase II clinical trial in patients with metastatic colorectal cancer due to insufficient efficacy at the dose level evaluated [52]. Promisingly, liposomal topotecan (FF-10850) was well tolerated in patients with ovarian cancer in a phase I dose-escalation study and is currently being investigated in expansion cohorts [53].

Solubilization and stabilization of CPTs can also be achieved using polymeric nanoparticulate systems, such as cyclodextrins and poly(lactic-co-glycolic acid) (PLGA). With a hydrophilic outer surface and a lipophilic cavity interior, cyclodextrins can interact with CPT to form noncovalent inclusion complexes. This complexed cyclodextrin–CPT has shown improved stability towards the retention of the active lactone against hydrolysis and increased water solubility [54,55]. Cyclodextrin–CPT complexes demonstrated comparable or higher in vitro potency in multiple cancer cell lines [56,57]. CRLX101 is an example of a cyclodextrin-based nanoparticle assembly of CPT, which has been clinically investigated [58,59]. PLGA formulations, owing to their acidic microclimate, have been demonstrated to stabilize the lactone form of CPTs [60]. A PLGA-based nanoparticle formulation of 9-nitrocamptothecin has shown higher cytotoxicity against a human ovarian cancer cell line and prolonged systemic half-life in rats compared with the free drug [61]. Nanoparticles can also achieve co-delivery of CPTs with other therapeutic agents as a combination therapy. A surface-modified cationic PLGA nanoparticle encapsulating CPT and curcumin has been shown to exert synergistic anti-cancer effects [62]. Overall, polymeric nanoparticle formulations have shown promise in enhancing efficacy, reducing toxicity, and therefore improving the therapeutic index of CPTs.

### 3.2. Antibody–Drug Conjugates

Among the various strategies that have been evaluated to enhance the selectivity of cancer chemotherapy, the use of monoclonal antibody–drug conjugates (ADCs) has proven to be particularly successful. As of the time of writing, thirteen ADCs have been approved by the US FDA, including two ADCs that deliver CPT derivatives: trastuzumab deruxtecan (T-DXd, Enhertu^®^) and sacituzumab govitecan (Trodelvy^®^) (Figure 3D,E) [18,19].

ADCs are comprised of an antibody that targets antigen-expressing tumor cells, a chemical linker, and a potent cytotoxic small-molecule payload. ADCs exert anti-cancer efficacy primarily through their entry into targeted cells via receptor-mediated endocytosis, releasing the payload after hydrolysis of the linker within the endosomes and/or lysosomes, and then transporting the payload to intracellular organelles associated with cytotoxic activity (e.g., within the cytoplasm or nucleus) [63]. The development of ADCs has primarily been focused on payloads that have demonstrated unsatisfactory efficacy and substantial toxicity when studied as unconjugated small molecules in clinical trials [64]. The side effects of most chemotherapeutic agents with a narrow therapeutic index are generally attributed to non-specific exposure to off-target tissues. The conjugation of these small molecules to an antigen-binding antibody often enhances the selectivity of drug delivery to targeted cells [65,66], significantly improving the therapeutic index of chemotherapeutics such as CPT derivatives.

Sacituzumab govitecan is an SN-38 ADC targeting trophoblast cell-surface antigen 2 (TROP-2), which received a breakthrough therapy designation from the FDA in 2021 for the treatment of relapsed patients with metastatic triple-negative breast cancer [67]. TROP2 was found in various human tumors [68], and TROP2 overexpression has been proposed to be associated with tumor progression in some types of cancers [69,70]. Comparing the safety profiles at the efficacious doses of irinotecan (the prodrug of SN-38) with those of sacituzumab govitecan, the ADC demonstrates significantly reduced grade 3+ diarrhea, which is one of the major DLTs of CPT-based chemotherapies [71]. Another approved ADC, trastuzumab deruxtecan (T-DXd), was developed by conjugating DXd to the previously approved HER-2 targeting monoclonal antibody (mAb) trastuzumab for the treatment of numerous HER2-expressing cancers. DXd is a derivative of exatecan, which exhibits highly potent anti-tumor activity in vitro but failed in clinical trials due to significant myelotoxicity [72,73,74]. T-DXd maintains high anti-cancer potency and possesses substantially reduced myelotoxicity, leading to FDA approval for the treatment of unresectable or metastatic HER2-positive breast cancer [75].

At the same antibody amount administered, a higher drug-to-antibody ratio (DAR) allows for more payload to be dosed [76]. CPT-based ADCs take advantage of the moderate cell-killing potency of CPT payloads, allowing a high DAR (around 8) and high-ADC doses (>5 mg/kg). With the proven success of the two CPT-based ADCs, more CPT derivatives have the potential to be explored as payload candidates for ADC therapeutics.

Some CPT-based ADCs are in late-phase development (Table 1). Patritumab deruxtecan (HER3-DXd) is an investigational DXd-conjugated ADC that has shown efficacy in patients with non-small-cell lung cancer (NSCLC) [77,78]. Although HER3-DXd is under inspection related to third-party manufacturing issues at the time of writing, no efficacy or safety concerns were reported in the FDA’s complete response letter. Datopotamab deruxtecan (Dato-DXd), comprising an anti-TROP2 mAb, demonstrated efficient DXd delivery into tumors and has been clinically investigated against multiple tumor types [79,80]. Dato-DXd showed significant improvement in progression-free survival compared with docetaxel in a phase III study and is currently under review by the FDA for application of use in NSCLC patients [81]. Although ADCs have several limitations, such as manufacturing challenges and dose-limiting toxicities, recent approvals in ADCs have marked them as one of the most successful classes of anti-cancer therapeutics [82,83,84].

## 4. Optimization Strategies Under Development

### 4.1. Inverse Targeting

#### 4.1.1. Small-Molecule CPT Derivatives

Antibodies or antibody fragments have been studied extensively to mitigate drug toxicity. Some are FDA-approved, such as an anti-digoxin immune Fab (Digifab^®^, BTG Pharmaceuticals, London, UK) for the treatment of life-threatening digoxin toxicity [85] and idarucizumab (Praxbind^®^, Boehringer Ingelheim, Ingelheim, Germany) for the management of serious bleeding by reversing the anticoagulant effects of dabigatran [86]. Similar utility has been shown for antibodies that bind chemotherapeutics with high affinity. Co-administration of anti-methotrexate Fab allowed for a 5-fold increase in the maximum tolerated dose of IP methotrexate in mice [87], co-administration of polyclonal anti-doxorubicin antibody improved the survival of mice treated with doxorubicin at toxic levels [88], and co-administration of a monoclonal anti-vinca antibody with toxic doses of vinca alkaloids led to 100% survival compared to vinca administration alone, which caused 70% mortality in mice [89].

The neutralizing activity of anti-drug antibodies has been exploited in a pharmacokinetic strategy termed “inverse targeting” to enhance the selectivity of regional chemotherapy [90,91]. In this approach, systemic administration of anti-drug antibodies is paired with regional administration of small-molecule chemotherapeutics (e.g., intraperitoneal chemotherapy for treatment of ovarian cancer). This strategy enables site-specific alterations in drug disposition, increasing the ratio of regional to systemic drug exposure. Unlike traditional, active targeting strategies, where a delivery system is employed to increase the efficiency of drug delivery to the desired site (e.g., tumor), inverse targeting attempts to enhance selectivity by decreasing drug delivery to sites associated with toxicity. Using topotecan as an example, the presence of anti-topotecan antibodies in the systemic circulation was hypothesized to lead to rapid binding of topotecan upon its absorption from the peritoneum, reducing systemic exposure of unbound drug, limiting the distribution of the drug to the bone marrow and gastrointestinal tract, which are key sites of topotecan toxicities [92]. Prior work demonstrated that a 2-fold decrease in body weight loss was observed with 30 mg/kg IP topotecan after co-administration of topotecan-binding antibodies in a mouse model of peritoneal cancer [93].

#### 4.1.2. Inverse Targeting to Enhance the Selectivity of ADC Therapy

Although ADC-mediated targeting to cancer cells improves the therapeutic selectivity of chemotherapeutics, off-target toxicity remains a significant concern [84]. Dose–efficacy and dose–toxicity curves for ADCs are often steep and overlapping, such that complete efficacy (i.e., elimination of targeted cancer cells) is not possible due to intolerable off-target toxicity. For example, in a phase I clinical trial of sacituzumab govitecan for the treatment of diverse epithelial cancers, a 25% increase in dose from 8 to 10 mg/kg nearly doubled the percentage (36% vs. 64%) of patients achieving a desirable response (e.g., stable disease or partial response) but also led to a greater-than 2-fold increase in the incidence rate of grade 3+ neutropenia (21% vs. 47%) [94]. In addition, although T-DXd is quickly becoming the drug of choice for the treatment of HER2-positive breast cancer, neutropenia and interstitial lung disease (ILD) are T-DXd-related adverse events that have been associated with some risks for treatment-related fatality [95]. Recent reviews pointed out that more than 90% of patients treated with ADCs exhibit at least one significant adverse effect [96]. Findings of several review articles also suggest ADC toxicities mainly arise from off-target delivery of the cytotoxic payload rather than target expression [84,97].

**Figure 5:**
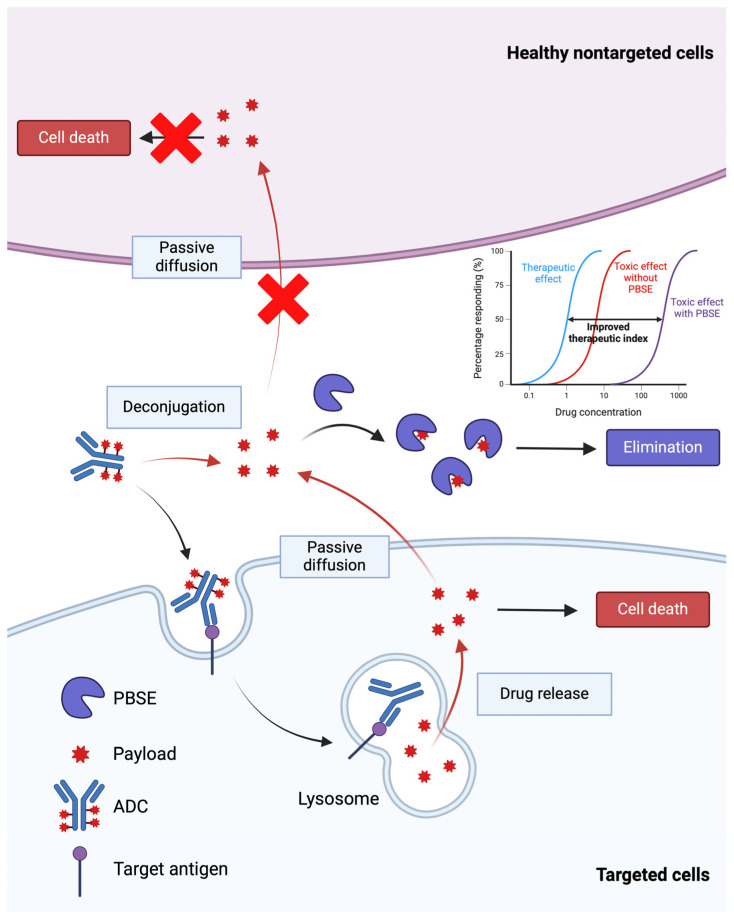
An illustration of the payload-binding selectivity enhancer (PBSE) strategy to improve the therapeutic index of ADCs. PBSEs, when co-administered with ADCs, are hypothesized to bind released payload and reduce the distribution of cytotoxic payload to sites of toxicity. Created with BioRender.com.

Based on differences in the putative primary mechanism of payload delivery to targeted cells (i.e., receptor-mediated endocytosis of ADC–antigen complexes) and payload delivery to non-targeted cells (i.e., cellular uptake via diffusion of released payload through plasma membranes), it was hypothesized that anti-payload antibody fragments may be employed to block payload entry into non-targeted cells while not negatively impacting payload delivery to targeted cells. This inverse targeting approach has recently shown promise in optimizing the therapeutic selectivity of ADC therapy [84,98,99]. Briefly, payload-binding antibody fragments (i.e., payload-binding selectivity enhancers or PBSEs) are co-administered with ADCs to bind, neutralize, and facilitate the elimination of released payload during ADC treatment (Figure 5). Antibody fragments such as Fab, scFv, and single-domain antibodies can serve as PBSEs and provide additional elimination pathways for bound payload through renal filtration. For example, when an anti-DXd PBSE is co-administered with a DXd ADC (e.g., T-DXd), the PBSE will rapidly bind the released DXd in the extracellular fluid, decreasing distribution to tissues associated with T-DXd toxicity and facilitating clearance by renal filtration and the elimination of PBSE-DXd complexes. Bordeau et al. have demonstrated that co-administration of an anti-MMAE PBSE reduced myelosuppression and weight loss following treatment with a MMAE-based ADC while not negatively affecting its anti-tumor efficacy in mice [98]. In addition, Nguyen et al. have shown that anti-DM4 PBSE were able to significantly increase mice tolerability of a DM4-based ADC, which enabled a reduction in tumor volume to undetectable levels and dramatic improvements in survival [99]. 8C2 Fab, a DXd-payload-binding PBSE fragment, selectively inhibits the cytotoxicity of DXd without affecting the cytotoxicity of T-DXd (unpublished work). In an in vitro cell culture, 8C2 Fab blocked the cell-killing effect of DXd, increasing the DXd concentration associated with 50% growth inhibition by 50-fold, while it did not decrease the cytotoxicity of T-DXd in NCI-N87 cells. In an in vivo xenograft mouse model, co-administration of 8C2 Fab to mice with 600 mg/kg T-DXd significantly decreased the %nadir body weight loss compared to results found in mice treated with T-DXd alone (12.8% versus 7.08%). In contrast, co-administration of 8C2 Fab with T-DXd (1 or 10 mg/kg) did not negatively impact the anti-tumor efficacy in mice bearing NCI-N87 xenograft tumors.

### 4.2. Application of Adjuvants to Increase Tumor Distribution of ADCs

#### 4.2.1. Anti-Idiotypic Distribution Enhancers

**Figure 6:**
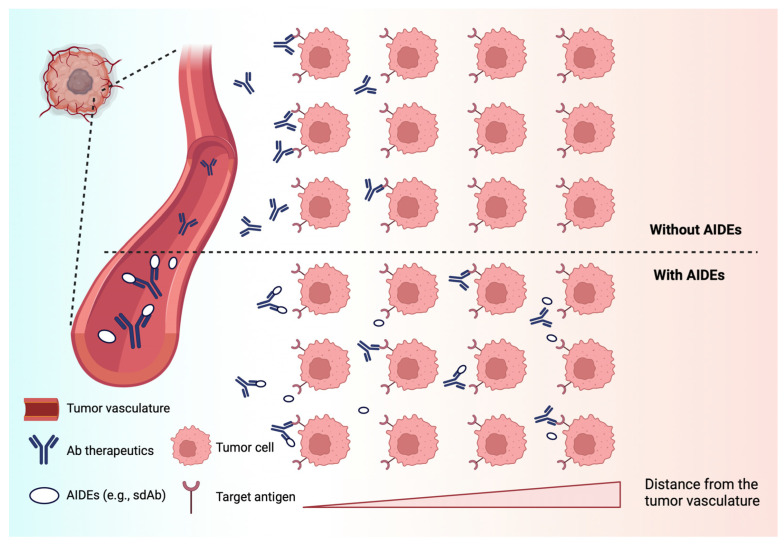
A schematic of the anti-idiotypic distribution enhancer (AIDE) strategy to improve the intra-tumoral delivery of antibody therapies. AIDEs bind to antibody therapeutics and act as competitive inhibitors. The AIDE-Ab complexes diffuse throughout the interstitial space and dissociate over time to allow for antibody binding to targeted tumor cells. Created with BioRender.com.

Current CPT-derivative-based chemotherapies, either approved or in development, are mostly indicated for solid tumors. The intra-tumoral distribution of large-molecule-based CPT therapeutics, such as ADCs, is often heterogeneous, and the depth of tumor penetration is limited. The abnormal physiology of solid tumors, including abnormal vascularization and high interstitial pressure, contributes to the poor distribution of large-molecule therapeutics [100,101,102]. For a targeted therapy, the antigen expression pattern in tumors also greatly impacts the distribution and subsequent efficacy of the drug. Recent work has shown that co-administration of anti-idiotypic agents (i.e., anti-idiotypic distribution enhancers, AIDEs), which transiently block the binding of antibody to tumor antigens (Figure 6), enable improved distribution and efficacy of anti-tumor antibodies, ADCs, and immunotoxins [102,103]. Co-administered AIDEs have been shown to improve the tumor distribution of mAb and enhance the efficacy of ADCs in mouse models.

#### 4.2.2. Co-Administration of “Naked” Antibody with ADCs

In addition to the use of anti-idiotypic agents, recent research has demonstrated that co-administration of ADCs with a ‘carrier’ dose of unconjugated antibody can mitigate the “binding site barrier” (BSB) within tumors to improve the tumor penetration and efficacy of ADCs [104]. Singh et al. have shown that the co-administration of trastuzumab increased the efficacy of trastuzumab emtansine (Kadcyla^®^, Genentech, Inc., South San Francisco, CA, USA) by competing with the ADC and partially blocking the receptors in a high-HER2-expression xenograft model [105]. They did not observe comparable benefits of the co-administration strategy in low-HER2-expression tumors, presumably due to less prominent BSB in low-HER2 tumors. To apply this co-administration strategy in low-target-expression tumor environment, Evans and Thurber designed a high-avidity, low-affinity carrier antibody that does not compete with the ADC in low-target-expression cells [106]. Additionally, under certain circumstances, the carrier dose of unconjugated antibody may efficiently block target-mediated uptake in normal tissues, allowing for enhanced tumor selectivity and reduced toxicity.

#### 4.2.3. Engineering Fragment–Drug Conjugates (FDCs)

Drug-specific properties like molecular size, binding affinity, valency, and dosing levels could also impact the distribution of these therapeutic agents in solid tumors. For example, use of antibody fragments or low-molecular-weight scaffolds may achieve deeper tumor penetration than full-size antibodies [107,108,109]. With recent advancements in protein engineering, several antibody fragments, such as antigen-binding fragment (Fab), single-chain variable fragment (scFv), and single-domain antibody (sdAb), are being designed and investigated as potential delivery vectors for cytotoxic payloads. The limited half-life of antibody fragments (within hours) can be overcome by the integration of high-affinity, reversible binding domains to carrier proteins like albumin, red blood cells, or IgG [110,111,112]. Dennis et al. examined the tumor distribution of anti-HER2 trastuzumab, trastuzumab-derived Fab (Fab4D5, 50 kDa), and ABD-coupled Fab4D5 (AB.Fab4D5, 52 kDa) [113]. They found that Fab4D5 rapidly but transiently appeared in the tumor at 2 h, whereas the presence of AB.Fab4D5 in the tumor was sustained beyond 24 h, similar to trastuzumab. In addition, comparable tumor deposition was achieved for both AB.Fab4D5 and trastuzumab; however, at peak tumor accumulation, AB.Fab4D5 was more uniformly distributed than trastuzumab. Nanobodies, also known as sdAbs, constitute the smallest antigen-binding fragments in nature, with a molecular weight of approximately 15 kDa [114]. Their deeper tissue penetration, short circulation time, and rapid renal clearance make them particularly suitable for tumor imaging applications, though their use as payload carriers has been limited due to quick elimination [115,116]. Recently, we demonstrated the therapeutic potential of conjugating DXd to a CEA-binding sdAb C17 (C17-DXd) or C17 coupled with ABD (C17ABD-DXd) in comparison to an anti-CEA antibody-conjugated DXd (10H9-DXd) (unpublished work). Our results indicated that C17-DXd, despite its lower in vitro potency and rapid plasma clearance (half-life of 0.47 h), exhibited more rapid tumor accumulation and greater tumor inhibition compared to C17ABD-DXd and 10H9-DXd. Moreover, the toxicity profile of C17-DXd, assessed by percentage body weight loss, showed improvement compared to 10H9-DXd and C17ABD-DXd. Despite its short plasma half-life of just minutes, C17-DXd maintained anti-tumor efficacy over one week without observable toxicity. Conversely, C17ABD-DXd, benefiting from prolonged systemic circulation due to the albumin binding domain (t1/2 = 35.9 h), showed PK and therapeutic profiles similar to the 10H9-DXd (t1/2 = 49.1 h), suggesting a balance between extended half-life and effective tumor targeting. Similarly, Nessler et al. found that compared to ADC, sdAb-conjugated DNA-alkylating agent (DGN549) exhibited increased tumor penetration and improved in vivo efficacy in prostate cancer models [117].

## 5. Conclusions

CPT and its derivatives have demonstrated substantial promise in cancer therapy owing to their potent inhibition of Topo I. Despite significant challenges related to solubility, stability, and toxicity, advances in drug delivery systems have significantly enhanced their clinical outcomes. Notably, liposomal formulations like liposomal irinotecan (Onivyde^®^) and antibody-drug conjugates such as trastuzumab deruxtecan (Enhertu^®^) and sacituzumab govitecan (Trodelvy^®^) have shown remarkable success by improving the targeted delivery of drugs to the site of action and reducing systemic toxicity. In addition, building upon the foundation of irinotecan and topotecan, new CPT derivatives via chemical modification are continuously being discovered. Ongoing clinical trials investigating combinations of CPT derivatives with other targeted therapies and immunotherapies hold promise for expanding their clinical utility. With the advancement in material and protein engineering, drug delivery systems have the potential to be further optimized. Additionally, innovative strategies such as inverse targeting and anti-idiotypic distribution enhancers are being investigated to further improve the therapeutic selectivity of antibody-mediated CPT chemotherapeutics.

## Figures and Tables

**Table 1:** Summary of camptothecin-based chemotherapeutics currently in phase II/III clinical trials.

Compound Name	Composition	Current Status	Indications
CRLX101	cyclodextrin-based polymer linked to CPT	Phase II	solid tumor
LY01610	liposomal irinotecan	Phase II	small-cell lung cancer
U3-1402 (patritumab deruxtecan)	anti-HER3-DXd ADC	Phase II	breast cancer, non-small-cell lung cancer
DS-1062 (datopotamab deruxtecan)	anti-Trop2-DXd ADC	Phase III	breast cancer, non-small-cell lung cancer
DS-6000 (raludotatug deruxtecan)	anti-CDH6-DXd ADC	Phase II/III	ovarian tumor, renal cell carcinoma
DS-7300 (ifinatamab deruxtecan)	anti-B7H3-DXd ADC	phase III	solid tumor
Abbv-400	anti-Met-CPT derivative ADC	Phase II	solid tumor
HS-20093	anti-B7H3-HS-9265 ADC	Phase II	solid tumor
SKB264	anti-TROP2-KL610023 ADC	Phase II	breast cancer, non-small-cell lung cancer
IBI343	anti-CLDN18.2-exatecan ADC	Phase II	gastric cancer
DB-1311	anti-B7H3-P1021 ADC	Phase I/II	solid tumor
